# Protective Effects of *Rhodiola Crenulata* Extract on Hypoxia-Induced Endothelial Damage via Regulation of AMPK and ERK Pathways

**DOI:** 10.3390/ijms19082286

**Published:** 2018-08-03

**Authors:** Pi-Kai Chang, I-Chuan Yen, Wei-Cheng Tsai, Tsu-Chung Chang, Shih-Yu Lee

**Affiliations:** 1 Graduate Institute of Medical Sciences, National Defense Medical Center, Taipei 11490, Taiwan; pencil8850@mail.ndmctsgh.edu.tw; 2 Division of Colon and Rectal Surgery, Department of Surgery, Tri-Service General Hospital, National Defense Medical Center, Taipei 11490, Taiwan; 3 School of Pharmacy, National Defense Medical Center, Taipei 11490, Taiwan; yenichuan@mail.ndmctsgh.edu.tw; 4 Graduate Institute of Aerospace and Undersea Medicine, National Defense Medical Center, Taipei 11490, Taiwan; j4418m@gmail.com; 5 Department of Biochemistry, National Defense Medical Center, Taipei 11490, Taiwan; tcchang@mail.ndmctsgh.edu.tw

**Keywords:** *Rhodiola crenulata*, hypoxia, endothelial cell, endothelial NOS, ERK, AMPK

## Abstract

*Rhodiola crenulata* root extract (RCE) has been shown to possess protective activities against hypoxia both in vitro and in vivo. However, the effects of RCE on response to hypoxia in the endothelium remain unclear. In this study, we aimed to examine the effects of RCE in endothelial cells challenged with hypoxic exposure and to elucidate the underlying mechanisms. Human umbilical vein endothelial cells were pretreated with or without RCE and then exposed to hypoxia (1% O_2_) for 24 h. Cell viability, nitric oxide (NO) production, oxidative stress markers, as well as mechanistic readouts were studied. We found that hypoxia-induced cell death, impaired NO production, and oxidative stress. These responses were significantly attenuated by RCE treatment and were associated with the activation of AMP-activated kinase and extracellular signal-regulated kinase 1/2 signaling pathways. In summary, we showed that RCE protected endothelial cells from hypoxic insult and suggested that *R. crenulata* might be useful for the prevention of hypoxia-associated vascular dysfunction.

## 1. Introduction

Endothelial dysfunction is associated with many diseases, including inflammatory bowel disease [1], atherosclerosis [2], and high-altitude pulmonary edema (HAPE) [3]. Under physiological conditions, the endothelium maintains vessel integrity, vascular permeability, and adhesion molecule expression, and regulates platelet activation, fibrinolysis, and coagulation [4]. When endothelial function is impaired, adhesion molecules and chemokines are overexpressed, which attracts immune cell migration and adhesion, subsequently leading to inflammatory responses and vasculitis [5]. Thus, endothelial dysfunction is widely recognized as an important therapeutic target for the treatment of vascular inflammatory diseases.

Oxidative stress is one of the most important factors driving hypoxia-induced endothelial dysfunction. For example, in patients with obstructive sleep apnea (OSA), hypoxia promotes endothelial dysfunction through bursts of reactive oxygen species (ROS) formation, cell apoptosis, and nitric oxide (NO) deficiency [6]. In pulmonary hypertension, hypoxia increases mitochondrial ROS generation and stimulates the activation of hypoxia-inducible factor (HIF)-1α-endothelin 1 (ET-1) axis [7] leading to hypoxic vasoconstriction, thereby increasing pulmonary pressure and right heart load. Similarly, in HAPE, hypoxia-induced oxidative stress can drive changes in vascular integrity and alveolar fluid balance, resulting in disease progression [8]. Targeting endothelial oxidative stress and NO bioavailability may be a promising therapeutic strategy to treat endothelial dysfunction in hypoxia, and anti-oxidative substances have shown beneficial effects on endothelial function in this setting [9]. 

The extracellular signal-regulated kinase 1/2 (ERK1/2) signaling pathway has been shown to be associated with endothelial survival during hypoxic exposure [10]. In contrast, blockade of ERK1/2 signal transduction results in endothelial apoptosis. Besides ERK, AMP-activated kinase (AMPK) is an important regulator of metabolic homeostasis and is essential for endothelial survival under hypoxia [10]. AMPK activation promotes endothelial nitric oxide synthase (eNOS) activity and preserves endothelial function [11]. Taken together, these results suggest that ERK1/2 and AMPK signaling pathways might also be therapeutic targets to preserve endothelial function under hypoxia.

*Rhodiola crenulata* is a common medicinal plant in Asian countries and has been reported to display significant anti-hypoxia, neuroprotective, anti-fatigue, and radio-protective activities [12]. We previously showed that *R. crenulata* extract (RCE) attenuates hypoxia-induced pulmonary injury and reverses ATPase endocytosis via ROS-AMPK-protein kinase C (PKC)ξ pathway [7,13]. RCE also restores the eNOS-NO-cyclic guanosine monophosphate (cGMP) relaxation pathway in hypobaric hypoxic rat heart [14]. In our recent study, RCE significantly improved cell death, NO defects, and oxidative stress in human umbilical vein endothelial cells (HUVECs) grown in high glucose conditions via the AMPK-AKT-eNOS-NO signaling pathways [15]. Thus, RCE might be beneficial for hypoxia-induced vascular endothelial dysfunction. However, the mechanisms underlying these actions remain unclear. In the present study, we established the effect of RCE on hypoxia-induced endothelial dysfunction and injury and explored the underlying mechanisms.

## 2. Results

### 2.1. Chemical Characterization of RCE

**Figure 1:**
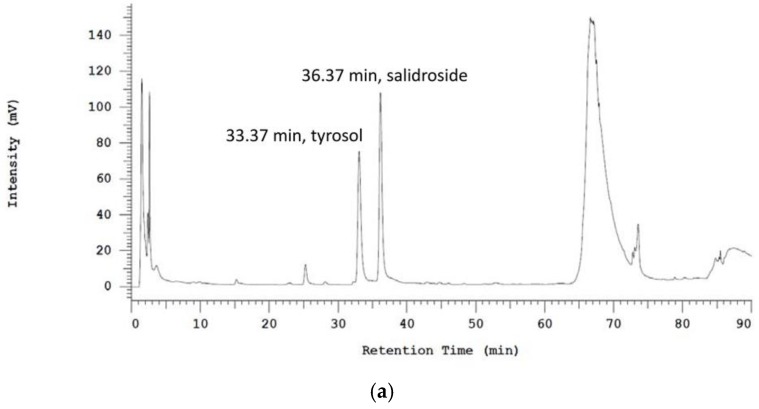

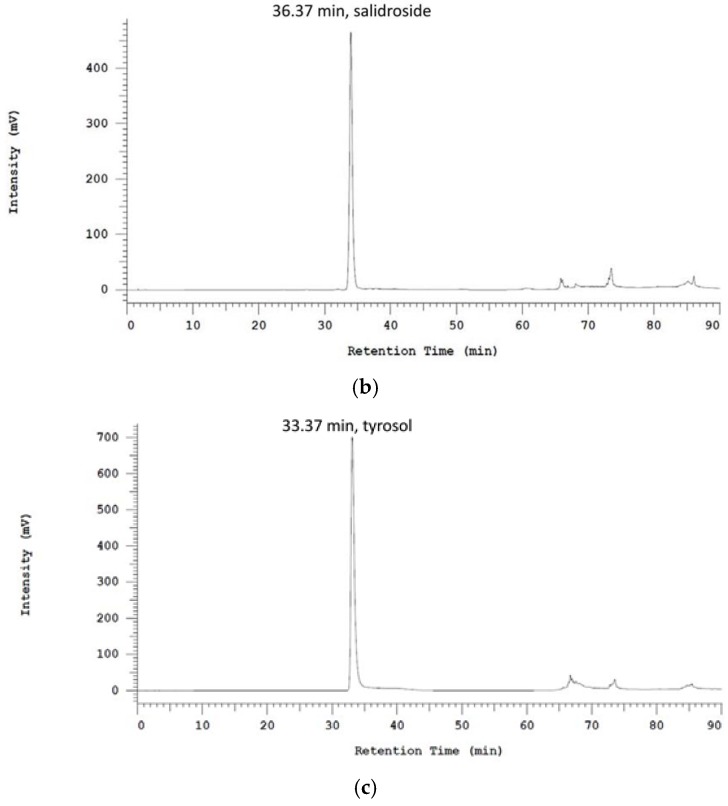
HPLC profiling data of *Rhodiola crenulata* root extract (**a**), salidroside (**b**), and tyrosol (**c**). The assay was performed as described in the Materials and Methods section. Detection was performed at a wavelength of 223 nm.

To assess the constituents of RCE, high performance liquid chromatography (HPLC) profiling of RCE, salidroside, and tyrosol was conducted. The calibration curve of tyrosol was linear within a range of 0.0625–1.0 mg/mL (R^2^ = 0.9998), and the calibration curve of salidroside was linear within a range of 0.125–1.0 mg/mL (R^2^ = 0.9986). HPLC analysis showed that RCE contained 2.43% salidroside and 1.1% tyrosol (Figure 1a–c).

### 2.2. RCE Protects against Hypoxia-Induced Endothelial Cell Death and Restores NO Production

**Figure 2:**
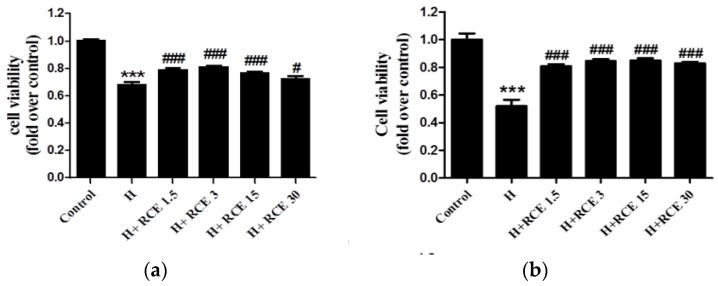

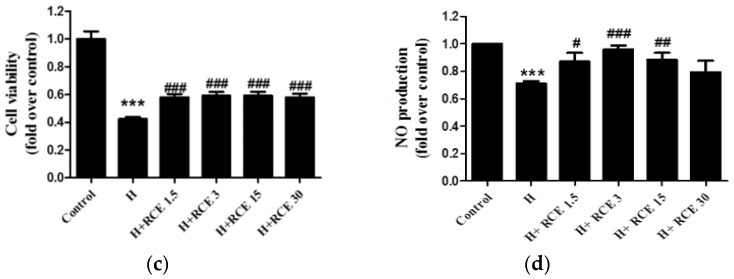
Effect of *Rhodiola crenulata* root extract (RCE) on cell viability (**a**) and nitric oxide (NO) production (**b**) in human umbilical vein endothelial cells (HUVECs) under hypoxic conditions. HUVECs were exposed to hypoxia (1% O_2_) with or without RCE treatment (μg/mL) for 24 (**a**), 48 (**b**), and 72 h (**c**). Cell viability was measured by Cell Counting Kit-8 (CCK-8 kit), and the supernatant (48 h) was collected for Griess assay (**d**). Results represent the mean ± SEM (*n* = 6). *** *p* < 0.001 versus control; # *p* < 0.05; ## *p* < 0.01; ### *p* < 0.001 versus hypoxia.

We first determined the effect of hypoxia on HUVECs. Exposure to hypoxia for 24 h significantly decreased the viability of HUVEC cells (0.68 ± 0.02-fold versus control, *p* < 0.001; Figure 2a), and this was significantly reversed by RCE treatment (0.79 ± 0.02, 0.81 ± 0.01, 0.76 ± 0.01, and 0.73 ± 0.02-fold over control at 1.5, 3.0, 15.0, and 30.0 μg/mL of RCE, respectively; Figure 2a). Similar effects were observed at both 48 (Figure 2b) and 72 h (Figure 2c). In addition, we found that NO production was significantly reduced in HUVECs during hypoxia (0.71 ± 0.02-fold versus control, *p* < 0.001; Figure 2b). NO production was restored by RCE treatment (0.87 ± 0.07, 0.96 ± 0.03, 0.88 ± 0.05, and 0.79 ± 0.08-fold over control at 1.5, 3.0, 15.0, and 30.0 μg/mL of RCE, respectively; Figure 2d). Both cell viability and NO production were reversed by RCE treatment, but not dose-dependently. 

### 2.3. RCE Protects Endothelial Cells from Oxidative Stress

**Figure 3:**
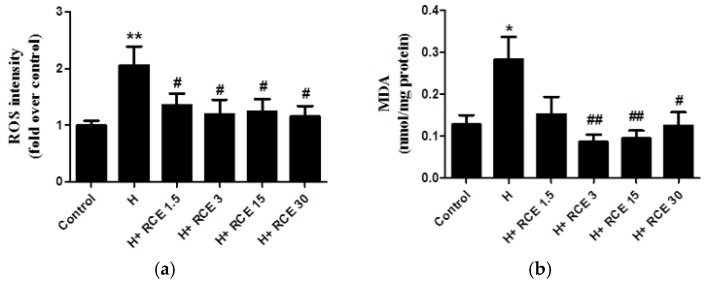
Effect of *Rhodiola crenulata* root extract on hypoxia-induced reactive oxygen species formation and lipid oxidation. HUVECs were pretreated with vehicle or RCE (μg/mL) and then exposed to hypoxia (1% O_2_) for 24 h. Afterwards, oxidative stress markers were detected with 2,7-dichlorofluorescein diacetate (DCFH-DA) (**a**) and MDA (**b**) assays as described in Materials and Methods. Results represent the mean ± SEM (*n* = 6). * *p* < 0.05; ** *p* < 0.01 versus control; # *p* < 0.05; ## *p* < 0.01 versus hypoxia.

To assess the anti-oxidant effects of RCE, we measured ROS and malondialdehyde (MDA) levels. Hypoxia significantly increased ROS levels (2.06 ± 0.33-fold compared with the control, *p* < 0.01; Figure 3a) as well as MDA levels (0.28 ± 0.05 nmol/mg protein, *p* < 0.05 compared with control; Figure 3b). Both hypoxia-induced ROS intensity (1.36 ± 0.20, 1.20 ± 0.26, 1.25 ± 0.22, and 1.16 ± 0.18-fold over control at 1.5, 3.0, 15.0, and 30.0 μg/mL of RCE; *p* < 0.05, respectively; Figure 3a) and MDA levels (0.09 ± 0.02, 0.10 ± 0.02, and 0.13 ± 0.03 nmol/mg protein at 3.0, 15.0, and 30.0 μg/mL of RCE; *p* < 0.01, *p* < 0.01, and *p* < 0.05, respectively; Figure 3b) were reduced by RCE treatment. 

### 2.4. RCE Restores Hypoxia-Impaired NO Production via AMPK-AKT-eNOS Signaling Pathway

**Figure 4:**
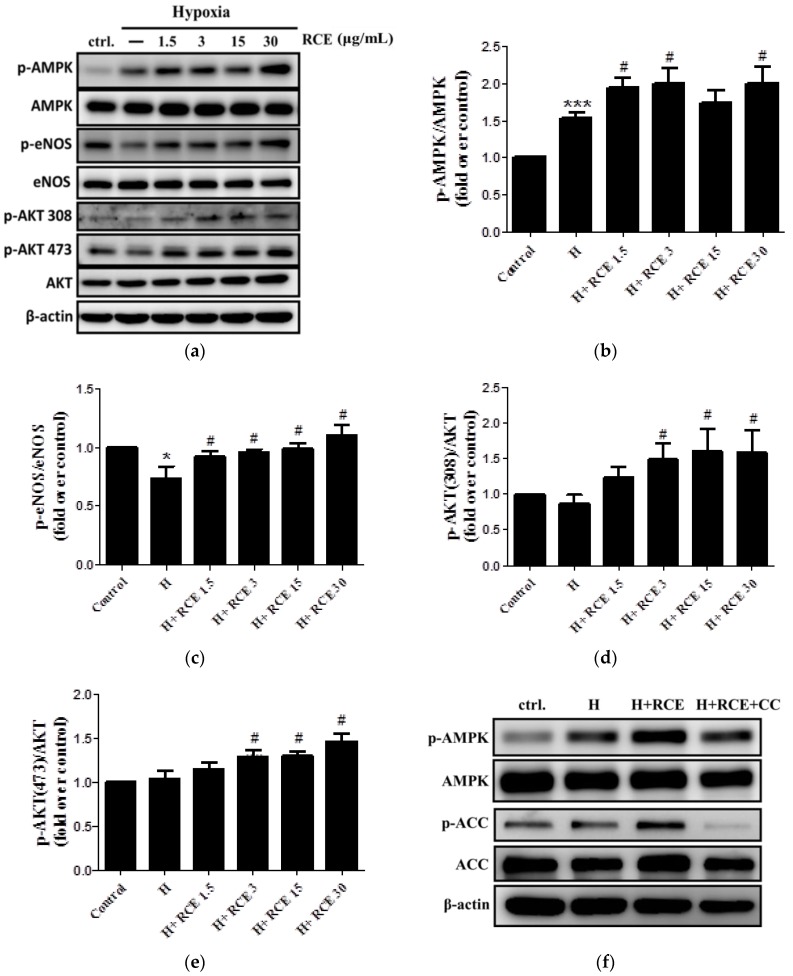

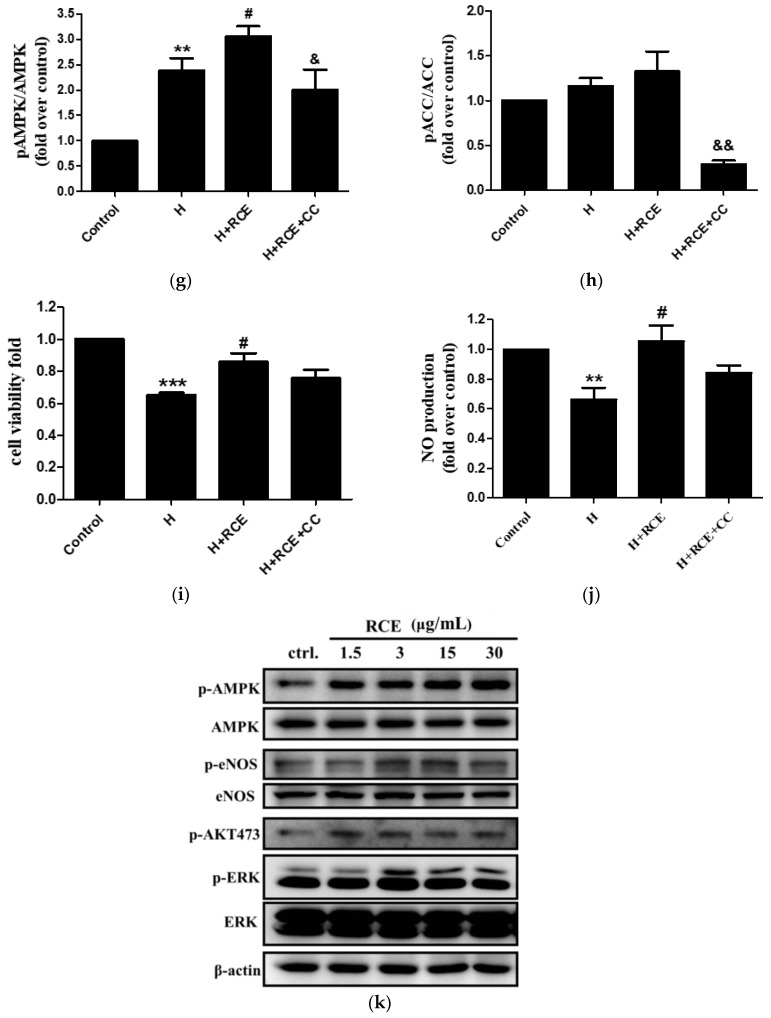
Effect of *Rhodiola crenulata* root extract on the AMP-activated kinase-AKT-endothelial nitric oxide synthase (AMPK-AKT-eNOS) axis in HUVECs under hypoxic conditions. Phosphorylation of AMPK (Thr172), eNOS (Ser1177), and AKT (Thr308 and Thr473) was analyzed by Western blotting (**a**). Quantitative analysis of the relative levels of p-AMPK (**b**), p-eNOS (**c**), p-AKT (Thr308; (**d**)), and p-AKT (Thr473; (**e**)) was conducted. The cells were further treated with AMPK inhibitor (CC; compound C; 2 μM) for 30 min before RCE treatment (3 μg/mL) and hypoxia exposure (1% O_2_) for 24 h. The effects of CC on p-AMPK and p-ACC were analyzed by Western blotting (**f**) and quantified ((p-AMPK; (**g**)) and (p-acetyl-coenzyme A carboxylase (ACC); (**h**)). β-actin served as a loading control. Cell viability was measured by using a CCK-8 kit (**i**), and the supernatant was collected for Griess assay (**j**). The cells were treated with RCE under normoxia (**k**). Results represent the mean ± SEM (*n* = 6). * *p* < 0.05; ** *p* < 0.01; *** *p* < 0.001 versus control; # *p* < 0.05 versus hypoxia; & *p* < 0.05 and && *p* < 0.01 versus hypoxic condition with RCE.

To investigate the regulatory mechanisms of RCE further, Western blotting was conducted. The protein level of p-AMPK (1.53 ± 0.07-fold compared with the control, *p* < 0.001; Figure 4a,b) was significantly increased, while that of p-eNOS (0.73 ± 0.10-fold compared with the control, *p* < 0.05; Figure 4a,c) was decreased under hypoxia. p-AKT at both Thr308 and Ser473 sites were unchanged (Figure 4a,d,e). RCE treatment increased p-AMPK levels (1.94 ± 0.13, 2.00 ± 0.20, 1.73 ± 0.17, and 1.99 ± 0.23-fold over control at 1.5, 3.0, 15.0, and 30.0 μg/mL of RCE; *p* < 0.05, *p* < 0.05, NS, and *p* < 0.05, respectively; Figure 4a,b), p-eNOS levels (0.92 ± 0.04, 0.96 ± 0.03, 0.99 ± 0.05, and 1.10 ± 0.09-fold over control at 1.5, 3.0, 15.0, and 30.0 μg/mL of RCE; *p* < 0.05, respectively; Figure 4a), as well as p-AKT at Thr308 (1.23 ± 0.16, 1.49 ± 0.23, 1.60 ± 0.31, and 1.58 ± 0.32-fold over control at 1.5, 3.0, 15.0, and 30.0 μg/mL of RCE; not significant (NS), *p* < 0.05, *p* < 0.05, and *p* < 0.05, respectively; Figure 4a,d) and Ser473 (1.16 ± 0.07, 1.30 ± 0.07, 1.31 ± 0.05, and 1.47 ± 0.09-fold over control at 1.5, 3.0, 15.0, and 30.0 μg/mL of RCE; NS, *p* < 0.05, *p* < 0.05, and *p* < 0.05, respectively; Figure 4a,e). In each case, RCE restored the levels to or above baseline levels. The maximum effect of RCE was observed at 3.0 μg/mL. To investigate the role of AMPK in the bioactivity of RCE further, we performed experiments with the AMPK inhibitor, Compound C. Compound C treatment slightly abolished the protective effects of RCE on hypoxia-induced changes in p-AMPK and p-ACC protein levels (Figure 4f–h), cell viability (*p =* 0.12, Figure 4i), and NO production (*p =* 0.07, Figure 4j).

### 2.5. RCE Protects Endothelial Cells from Hypoxia-Induced Apoptosis 

**Figure 5:**
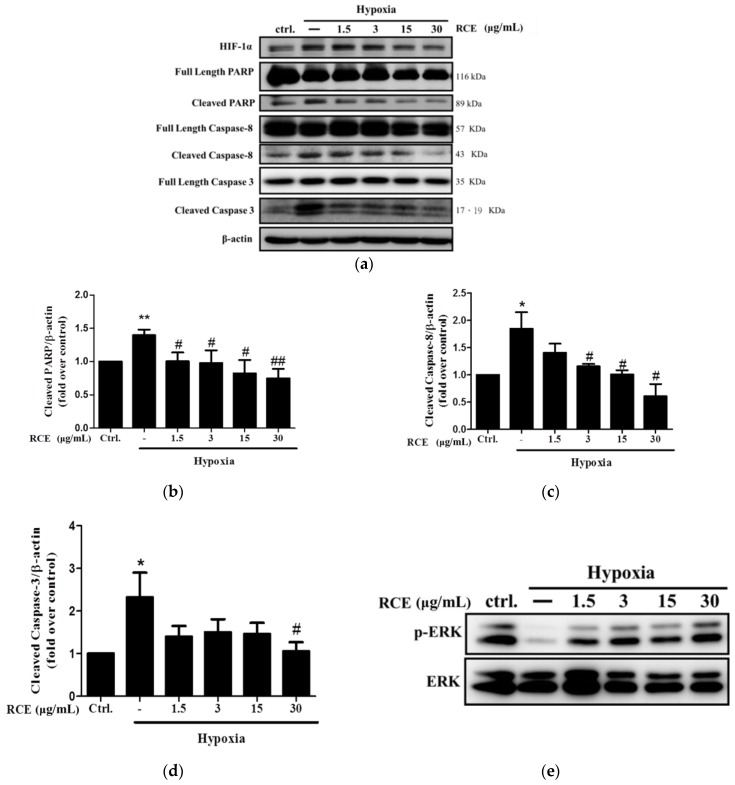

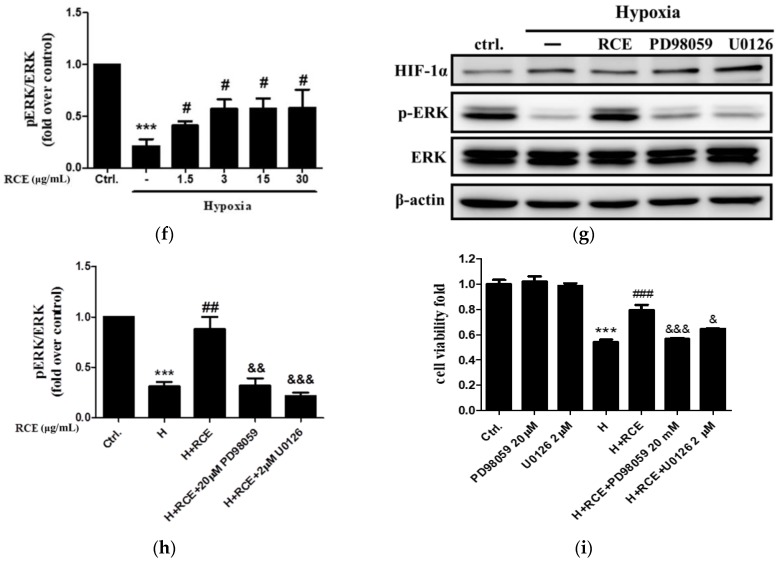
Effect of *Rhodiola crenulata* root extract on hypoxia-induced endothelial cell apoptosis and extracellular signal-regulated kinase 1/2 (ERK1/2) signaling pathway. After exposure to hypoxia (1% O_2_) for 24 h with or without RCE treatment, the protein levels of PARP, caspase-3, -8 (**a**), and p-ERK/ERK (**e**) were analyzed by Western blotting. The quantitative analysis of cleaved PARP (**b**), cleaved caspase-8 (**c**), cleaved caspase-3 (**d**), and p-ERK/ERK (**f**) were conducted. The cells were further treated with ERK inhibitor (PD98059 20 μM or U0126 2 μM) for 30 min, followed by incubation with RCE (3 μg/mL) and exposed to hypoxia (1% O_2_). The effects of ERK inhibitors on p-ERK/ERK (**g**,**h**) and cell viability (**i**) were determined. Results represent the mean ± SEM (𝑛 = 3). * *p* < 0.05, ** *p* < 0.01, and *** *p* < 0.001 versus control; # *p* < 0.05, ## *p* < 0.01, and ### *p* < 0.001 versus hypoxia; & *p* < 0.05, && *p* < 0.01, and &&& *p* < 0.001 versus hypoxic condition with RCE.

To examine the effects of RCE on hypoxia-induced apoptosis, the levels of crucial apoptotic proteins were analyzed. The levels of cleaved poly ADP ribose polymerase (PARP) (1.40 ± 0.09-fold over control, *p* < 0.01, Figure 5a,b), caspase 8 (1.85 ± 0.30-fold over control, *p* < 0.05, Figure 5a,c), and caspase 3 (2.32 ± 0.58-fold over control, *p* < 0.05, Figure 5a,d) were significantly increased by hypoxia. However, these effects were almost completely reversed by RCE treatment (Figure 5a–d). To clarify the pro-survival signaling pathway by which RCE acts in HUVECs, the phosphorylation of the pro-survival protein ERK was quantified. Consistently, we found that p-ERK at Thr202/Tyr204 (0.21± 0.07-fold over control at *p* < 0.001, Figure 5e,f) was significantly decreased under hypoxic conditions, and this was restored by RCE treatment (Figure 5e,f). To confirm the role of ERK on hypoxia-induced apoptosis further, the effects of the ERK inhibitors, U0126 and PD98059, were studied. Both ERK inhibitors significantly abolished phosphorylation of ERK (Figure 5g,h) and prevented the protective effects of RCE on endothelial cell death (Figure 5i). These findings indicate that RCE protects the cells from hypoxic insult in an ERK-dependent manner.

## 3. Discussion

**Figure 6:**
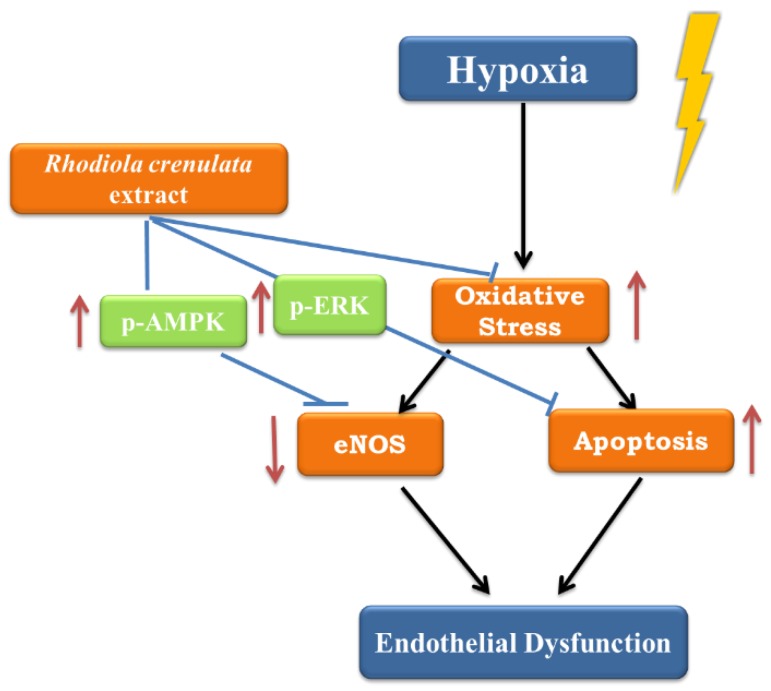
Proposed mechanisms of *Rhodiola crenulata* root extract on hypoxia-induced endothelial dysfunction. Arrows: increase; T-bar: attenuation; 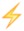: hypoxic stimulation.

Endothelial cells are exposed to hypoxic environments in several physiopathological conditions, such as myocardial infarction, atherosclerosis, pulmonary hypertension, and high altitude [16]. Hypoxia alters the production of endothelial vasoactive mediators such as NO and ET-1, induces oxidative stress, and decreases endothelial cell viability. These changes result in vascular inflammation and damage [17] and are associated with cardiovascular morbidity and mortality [18]. In the present study, we examined RCE as a protective agent against endothelial response to hypoxia. RCE significantly prevented cell death, restored NO deficiency, and reduced oxidative stress markers under hypoxic condition. These protective effects of RCE against hypoxic insult were dependent on multiple mechanisms, including anti-oxidant activity, AMPK-AKT-eNOS, and ERK signaling pathways (Figure 6).

Oxidative stress plays an indispensable role in the pathogenesis of endothelial dysfunction and cardiovascular diseases [19]. In the present study, RCE attenuated both hypoxia-induced ROS production and lipid oxidation. These findings indicate that RCE can block hypoxia-induced endothelial oxidative stress. In agreement with these findings, previous studies reported that salidroside, the bioactive component in *Rhodiola* species, and its aglycone tyrosol act as ROS scavengers and induce antioxidant enzyme activities in different cell models [13,20,21,22]. Based on these findings, the effect of RCE on hypoxia-induced oxidative stress may well reflect the antioxidant properties of its constituents. Endothelial oxidative stress impairs eNOS activity and reacts with NO to form peroxynitrite, subsequently leading to a vicious cycle of endothelial injury [23]. This plays a central role in the initiation of hypoxia-induced vascular disorders, such as cardiovascular diseases [24] and HAPE progression [3]. In our previous studies, we have shown that RCE can significantly attenuate hypoxia-induced oxidative stress and pulmonary edema [7,13]. The results of our present study may partially explain these anti-altitude effects of *R. crenulata* via its actions on the endothelium, with RCE preventing the development of the condition by reducing ROS formation, thereby improving the bioavailability of endothelial NO. Although the present study focused on oxidative stress and NO pathway, endothelial cells respond to stress through changes in various vasoactive and mitogenic factors, including vascular endothelial growth factor (VEGF) and ET-1 [16], which can be harmful themselves but provide feedback regulation of the NO pathway. For example, ET-1 inhibits eNOS activity through the PKC pathway [25]. In future studies, it would be interesting to investigate the effect of RCE on other vasoactive factors in endothelial cells after hypoxic challenge.

Endothelial apoptosis contributes to many features of endothelial dysfunction, and apoptotic vascular endothelial cells have been observed in patients with cardiovascular disease [26]. In this study, we showed that RCE treatment significantly decreased protein markers of apoptosis, indicating that RCE protects against hypoxia-induced endothelial apoptosis. Our results are consistent with those of a previous study, wherein the RCE component salidroside was shown to prevent endothelial cell hypoxia induced by cobalt chloride [27]. Our data also showed that the protective effects of RCE against hypoxic exposure occurred in an ERK-dependent manner. Activation of ERK has been shown to promote endothelial cell survival by suppressing pro-apoptotic proteins, such as caspase 3 and Bad [10]. RCE (30 μg/mL) appeared to inhibit the beneficial effects of RCE treatment, as opposed to the effects on AKT activation. Thus, it can be suggested that a higher dosage of RCE may affect certain cellular activities. A dose-independent activity has been reported commonly in natural products [28]. In agreement with the mechanisms studied here, hypoxia has been reported to induce endothelial cell apoptosis through endoplasmic reticulum (ER) stress [29]. The effects of RCE on these pathways should be the basis of future studies.

The impairment of eNOS-NO system is a hallmark of endothelial dysfunction and has been strongly linked to the initiation and development of cardiovascular diseases, such as atherosclerosis and coronary heart disease [30]. This is also a feature of hypoxia. In patients with OSA, low plasma NO levels and eNOS activity have been observed [31]. In this study, we observed that RCE rescued endothelial NO production and signaling via AMPK-AKT-eNOS axis under hypoxia. This is consistent with our previous studies demonstrating that RCE counteracts the effect of hypobaric hypoxia in rat heart through rebalancing NO and arginase 1 signaling pathways [14], and protects HUVECs from high glucose challenge via AMPK-AKT-eNOS-NO signaling pathway [15]. In addition, NO has been reported to maintain cytoskeletal structure and suppress apoptosis in endothelial cells [32,33]. The effects of RCE that we observed on hypoxia-induced endothelial apoptosis could also be partial via recovery of NO signaling pathway. Therefore, *R. crenulata* might be beneficial for many vascular diseases related to hypoxic damage and NO dysfunction in the endothelium. In the case of OSA, RCE may offer additional benefits because OSA is associated with poor glycemic control [34], and RCE has shown protective effects in this context [15,35]. Based on this finding, RCE might be considered a potential agent for vascular disorders in diabetic patients with OSA syndrome. However, further clinical studies are required to clarify this. Hypoxia triggered endothelial AMPK activation, which is mainly mediated angiogenesis by PI3K/AKT signaling pathway [36]. AMPK is also essential for HIF-1-VEGF axis-mediated NO production in endothelial cells [37]. These findings imply that the effect of RCE on AMPK-AKT-eNOS axis might be VEGF dependent. It would be interesting to investigate the efficacy of RCE on VEGF signal transduction. 

## 4. Materials and Methods

### 4.1. Preparations and HPLC Analysis of RCE 

RCE was prepared as previously described [12]. Briefly, dry root powder (2.0 kg) was extracted with 95% EtOH (10 L × 2) and then refluxed with 95% EtOH (10 L × 1). Evaporation of the solvent under reduced pressure yielded 320.24 g of crude extract (RCE, 16.0% yield). The active markers, salidroside and tyrosol, were isolated by the School of Pharmacy, National Defense Medical Center. The constituents of RCE were analyzed using a Hitachi instrument with an L-7100 series quaternary gradient pump (Hitachi, Japan) and a diode array detector (L-7455) linked to a Hitachi LaChrom software data handling system (D-7000 Multi-HSM-Manager, Merck–Hitachi, Tokyo, Japan). Reverse-phase separations were carried out using a Lichro CART^®^ RP-18e column (4.0 × 250 mm i.d., 5μm; Merck, Darmstadt, Germany). Reversed-phase HPLC was performed by using a water–acetonitrile gradient as the mobile phase. The identity of constituents was also confirmed with a photodiode array detector and compared with known UV spectra of standards at 223 nm. 

### 4.2. Cell Culture

HUVECs are widely used to study endothelial injury in vitro [15]. HUVECs (Lifeline Cell Technology, Walkersville, MD, USA) were cultured in VascuLife complete medium (Lifeline Cell Technology) at 37 °C in a humidified 5% CO_2_ incubator. The cells at passages 4–10 were used in these experiments. For hypoxic exposure, the cells were maintained in VascuLife basal medium supplemented with 2% fetal bovine serum and exposed to hypoxic conditions (1% O_2_) in a humidified 5% CO_2_ hypoxic chamber (Astec, Fukuoka, Japan). 

### 4.3. Cell Viability Assay

Cell viability was determined as previously described [12]. Briefly, HUVECs (5 × 10^3^ cells/well) were seeded into 96-well plates overnight and then exposed to hypoxia (1% O_2_) for 24 h. Cell Counting Kit-8 reagent (10%; Dojindo, Japan) was then added to the cells, and after 2 h, the absorbance was measured at 450/650 nm using a spectrophotometer (Molecular Devices, Sunnyvale, CA, USA).

### 4.4. Measurement of Nitrite

Nitrite was determined as previously described [38]. HUVECs (3.5 × 10^5^ cells/well) were seeded into 6-well plates. The supernatant was collected (85 μL) and added to Griess reagent (100 μL) (BioVision Inc., Mountain View, CA, USA). The absorbance was measured at 540 nm using an EIA Reader (Molecular Devices, Sunnyvale, CA, USA).

### 4.5. Malondialdehyde Analysis

MDA levels were quantified by using a commercial kit (Cayman Chemical Co., Ann Arbor, MI) as described previously [15]. Briefly, the cells were lysed in RIPA lysis buffer and centrifuged at 1600× *g*. The supernatant (100 µL) was mixed with 100 µL sodium dodecyl sulfate (SDS) and 4 mL color reagent containing 8.1% thiobarbituric acid. The samples were boiled for 1 h and then centrifuged at 1600× *g* for 10 min. The absorbance of the supernatant was measured at 535 nm using a spectrophotometer (Molecular Devices, Sunnyvale, CA, USA).

### 4.6. Measurement of Intracellular ROS

Intracellular ROS levels were assayed using a fluorescent probe, 2,7-dichlorofluorescein diacetate (DCFH-DA), as described previously [15]. In brief, the cells (10^4^ cells/well) were seeded in a 96-well plate overnight and then exposed to hypoxia (1% O_2_) for 24 h. The original medium was removed and replaced with 10 µM DCFH-DA in serum-free medium for 15 min at 37 °C. The cells were then lysed with RIPA buffer and centrifuged at 1600× *g* for 1 min. The supernatant was collected, and the excitation and emission wavelengths at 480 and 530 nm were measured using a fluorescence microtiter plate reader (POLARstar Galaxy; BMG Labtechnologies, Offenburg, Germany). 

### 4.7. Western Blot Analysis

Western blot analysis was carried out as described previously [39]. Briefly, the cells were harvested in 0.1 mL of RIPA lysis buffer. Total protein was quantified using a BCA protein assay kit (Pierce, Rockford, IL, USA), separated by 8–12% SDS-polyacrylamide gel electrophoresis, and transferred onto polyvinylidene difluoride membranes (Millipore, Bedford, MA, USA). Immunoblotting was performed using primary and secondary antibodies given in Table 1. The signals were visualized using an enhanced chemiluminescence kit (ECL, Amersham Biosciences, Buckinghamshire, UK), followed by exposure of the blots to X-ray film. The relative density of band was determined densitometrically and normalized with respect to β-actin, AMPK, eNOS, AKT, ACC, or ERK, using the Image J software (version 1.50a).

### 4.8. Statistical Analysis

All data shown represent the mean ± S.E.M. Significant differences among group means were determined by one-way ANOVA, followed by Bonferroni post-hoc test using the IBM SPSS Statistics version 22 (IBM^®^ SPSS^®^ Statistics 22, Armonk, NY, USA). Significance was accepted when *p* < 0.05.

## 5. Conclusions

Our findings demonstrate that RCE protects endothelial cells from hypoxic insults via multiple mechanistic pathways and suggest that *R. crenulata* might be beneficial for the treatment of hypoxia-associated vascular diseases.

## Figures and Tables

**Table 1:** Antibodies used for Western blotting.

Type	Antigen	Manufacturer	Dilution
Primary antibody	eNOS	Santa Cruz, CA	1:1000
Primary antibody	p-AKT (Thr308 and Ser473)	Santa Cruz, CA	1:1000
Primary antibody	AKT	Santa Cruz, CA	1:1000
Primary antibody	β-actin	Chemicon, Temecula, CA	1:1000
Primary antibody	p-eNOS (Ser1177)	Cell Signaling Tech.	1:1000
Primary antibody	AMPK	Cell Signaling Tech.	1:1000
Primary antibody	p-AMPK (T172)	Cell Signaling Tech.	1:1000
Primary antibody	ACC	Cell Signaling Tech.	1:1000
Primary antibody	p-ACC	Cell Signaling Tech.	1:1000
Primary antibody	PARP	Cell Signaling Tech.	1:1000
Primary antibody	Caspase 3	Cell Signaling Tech.	1:1000
Primary antibody	Caspase 8	Cell Signaling Tech.	1:1000
Primary antibody	ERK	Cell Signaling Tech.	1:1000
Primary antibody	p-ERK	Cell Signaling Tech.	1:1000
Secondary antibody	Anti-goat IgG-HRP	Santa Cruz, CA	1:10,000
Secondary antibody	Anti-rabbit IgG-HRP	GeneTex	1:10,000
Secondary antibody	Anti-mouse IgG-HRP	Jackson	1:10,000
